# Zn-induced electron-rich Sn catalysts enable highly efficient CO_2_ electroreduction to formate†

**DOI:** 10.1039/d3sc02790b

**Published:** 2023-07-10

**Authors:** Xingxing Tan, Shunhan Jia, Xinning Song, Xiaodong Ma, Jiaqi Feng, Libing Zhang, Limin Wu, Juan Du, Aibing Chen, Qinggong Zhu, Xiaofu Sun, Buxing Han

**Affiliations:** a Beijing National Laboratory for Molecular Sciences, Key Laboratory of Colloid and Interface and Thermodynamics, Center for Carbon Neutral Chemistry, Institute of Chemistry, Chinese Academy of Sciences Beijing 100190 P. R. China sunxiaofu@iccas.ac.cn hanbx@iccas.ac.cn; b School of Chemical Sciences, University of Chinese Academy of Sciences Beijing 100049 P. R. China; c College of Chemical and Pharmaceutical Engineering, Hebei University of Science and Technology Shijiazhuang 050018 P. R. China; d Shanghai Key Laboratory of Green Chemistry and Chemical Processes, School of Chemistry and Molecular Engineering, East China Normal University Shanghai 200062 P. R. China

## Abstract

Renewable-energy-driven CO_2_ electroreduction provides a promising way to address the growing greenhouse effect issue and produce value-added chemicals. As one of the bulk chemicals, formic acid/formate has the highest revenue per mole of electrons among various products. However, the scaling up of CO_2_-to-formate for practical applications with high faradaic efficiency (FE) and current density is constrained by the difficulty of precisely reconciling the competing intermediates (*COOH and HCOO*). Herein, a Zn-induced electron-rich Sn electrocatalyst was reported for CO_2_-to-formate with high efficiency. The faradaic efficiency of formate (FE_formate_) could reach 96.6%, and FE_formate_ > 90% was maintained at formate partial current density up to 625.4 mA cm^−1^. Detailed study indicated that catalyst reconstruction occurred during electrolysis. With appropriate electron accumulation, the electron-rich Sn catalyst could facilitate the adsorption and activation of CO_2_ molecules to form a 
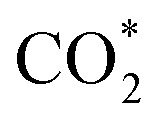
 intermediate and then promoted the carbon protonation of 
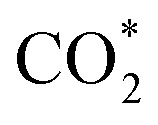
 to yield a HCOO* intermediate. Afterwards, the HCOO* → HCOOH* proceeded *via* another proton-coupled electron transfer process, leading to high activity and selectivity for formate production.

## Abstract 2

A Zn-induced electron-rich Sn catalyst was constructed *via* a rational electronic structure manipulation strategy. It showed high efficiency for CO_2_ electroreduction to formate.

## Graphical abstract

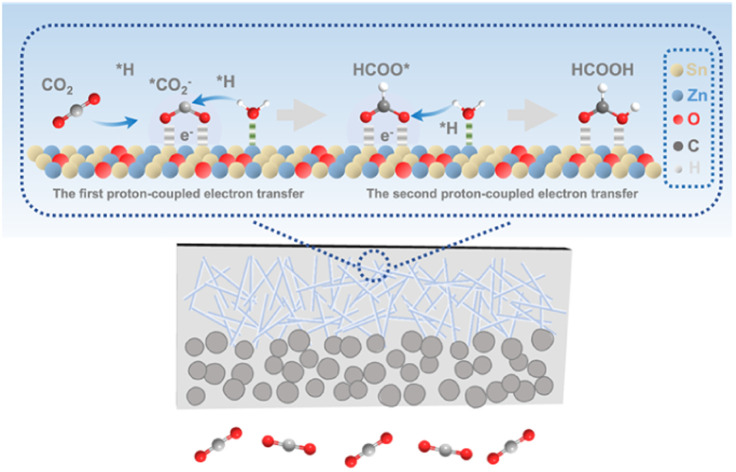

## Introduction

The electrochemical CO_2_ reduction reaction (eCO_2_RR) to value-added chemicals and fuels utilizing renewable electricity offers a sustainable route to offset the extra carbon footprint.^1–3^ However, this reaction is highly energetic and unfavorable, and a thermodynamic potential of −1.90 V *vs.* the standard hydrogen electrode (SHE) is needed to activate CO_2_ to *CO_2_^−^.^4^ Due to the competing hydrogen evolution reaction (HER) and the similarity of the redox potentials (from −0.2 to 0.6 V *vs.* SHE) for all the subsequent proton-assisted processes,^5,6^ eCO_2_RR pathways generally result in a mixture of products. Different studies have aimed to understand the fundamental factors that control the product selectivity, including optimizing catalytic conditions and developing novel catalysts.^7–12^ The adsorption behavior of key intermediates is strongly dependent on the geometric and electronic structure of the catalyst surface.^3,13–16^ Although some breakthroughs have been made in improving the selectivity for a desired product, it is still in the initial stage of meeting the demands of scaling up the eCO_2_RR for practical applications with high faradaic efficiency (FE) and current density.

Among various CO_2_-derived products, formic acid/formate presents the highest revenue per mole of electrons.^17,18^ Formic acid is a commonly used feedstock in the pharmaceutical and chemical industries.^17^ In addition, with its impressive energy density and convenient transportation, formic acid is also extensively studied as a promising hydrogen carrier for fuel cells.^19,20^ In the reaction pathway of the eCO_2_RR to formate, activated CO_2_ undergoes a proton-coupled electron transfer (PCET) process to give the HCOO* intermediate and then experiences another transfer to reduce HCOO* to HCOO^−^.^21^ This combination of processes is generally related to the intrinsic properties of the catalyst. Sn is a promising candidate toward formic acid/formate because of its favorable binding energy for HCOO*.^22,23^ However, Sn also shows a certain binding energy to *COOH, resulting in the generation of a CO by-product.^24^ A promising approach to direct the eCO_2_RR over Sn to the HCOO* pathway is to introduce metallic heteroatom doping to construct Sn-based catalysts, which can manipulate the electronic structure of the catalysts to facilitate both the formation and stabilization of the HCOO* intermediate.^25–27^ Notably, Sn-based catalysts may undergo structural evolution during the electrochemical process, and then the actual active sites will be created to trigger an efficient catalytic reaction. Therefore, it is significant to reveal the structural evolution of Sn-based catalysts and reveal active sites to achieve efficient CO_2_ reduction.^22^

Herein, we have constructed a Sn–Zn electrocatalyst (Sn–Zn–O_*x*_) for the eCO_2_RR to formate. It exhibited a maximum faradaic efficiency for formate (FE_formate_) of 96.6% and >90% FE_formate_ was maintained with a partial current density of formate (*j*_formate_) up to 625.4 mA cm^−1^. Experimental and density functional theory (DFT) calculations revealed that the reconstructed Sn sites could facilitate the adsorption and activation of CO_2_ molecules to form a 
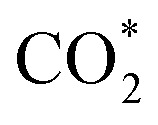
 intermediate and then promoted the carbon protonation of 
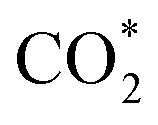
 to intermediate HCOO*. Successively, HCOO* absorbed on Sn–Zn–O_*x*_ enabled H* to adsorb and react with it more accessibly, which could lower the thermodynamic barrier in the second PCET process for the formation of formate.

## Results and discussion

**Fig. 1:**
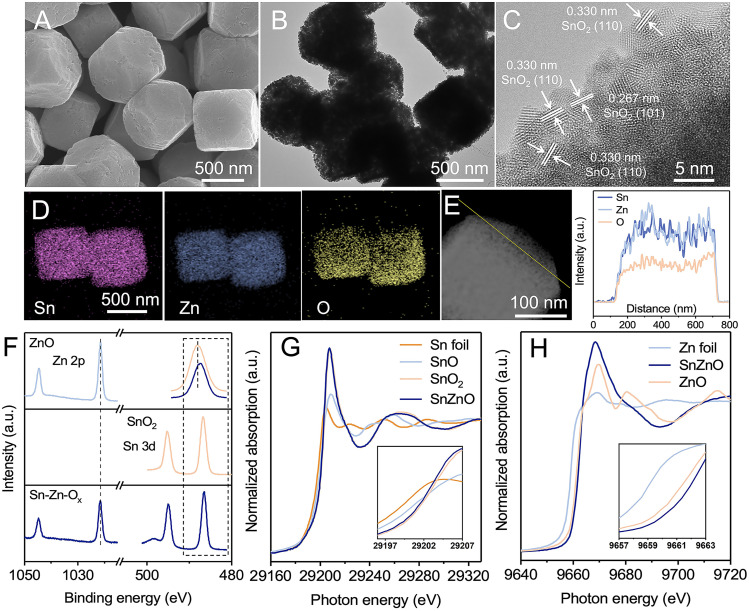
Morphology and structure characterization of the as-prepared catalysts. (A) SEM image, (B) TEM image, and (C) HR-TEM image of Sn–Zn–O_*x*_. (D) EDS mapping and (E) line scan of Sn–Zn–O_*x*_. (F) XPS spectra of Sn 3d and Zn 2p orbits. (G) Sn K-edge XANES spectra of Sn–Zn–O_*x*_, SnO_2_, SnO reference (SnO-ref), and Sn foil (Sn-ref). (H) Zn K-edge XANES spectra of Sn–Zn–O_*x*_, ZnO, and Zn foil (Zn-ref).

The Sn–Zn–O_*x*_ nanocomposites were synthesized using a facile coprecipitation method followed by pyrolyzing at 500 °C for 2 h in an argon atmosphere. The Sn/Zn atomic ratio of the obtained catalysts was 0.85, which was determined by inductively coupled plasma optical emission spectrometry (ICP-OES). Scanning electron microscopy (SEM) and transmission electron microscopy (TEM) images show that Sn–Zn–O_*x*_ composites displayed a uniform truncated cubic morphology with edge lengths of about 500 nm (Fig. 1A, B, S1 and S2†). The truncated cube was composed of smaller nanoparticles and was rich in mesopores with a massive pore volume of 6.3 nm, which favored the exposure of more active sites during the eCO_2_RR. Only a broad diffraction peak can be observed for Sn–Zn–O_*x*_ in the X-ray diffraction (XRD) patterns (Fig. S3†), perhaps due to the small size of the granules. The high-resolution TEM (HRTEM) image shows a lattice spacing of 0.267 nm and 0.330 nm, corresponding to the (110) and (101) planes of SnO_2_,^28^ respectively (Fig. 1C). The energy dispersive X-ray (EDX) elemental mapping and line-scan analysis confirmed that Sn, Zn, and O elements were distributed uniformly over the entire architectures (Fig. 1D and E). Using the same method, we also synthesized ZnO and SnO_2_ for comparison. Their SEM and TEM images are shown in Fig. S4 and S5.†

X-ray photoelectron spectroscopy (XPS) and X-ray absorption spectroscopy (XAS) were then performed to reveal the composition and structural information of Sn–Zn–O_*x*_. As illustrated in Fig. 1F, the Zn 2p_3/2_ and Zn 2p_1/2_ peaks of Sn–Zn–O_*x*_ at 1021.63 eV and 1044.79 eV are slightly higher than those of Zn^2+^ in ZnO. The Sn 3d_5/2_ and Sn 3d_3/2_ peaks of Sn–Zn–O_*x*_, located at 486.52 eV and 494.97 eV, shifted to a lower binding energy by about 0.21 eV compared with SnO_2_. The opposite shifts for Zn 2p and Sn 3d orbital peaks indicate the interaction between Zn and Sn, resulting in a modified electronic structure.^29^ O 1s spectra were also recorded and are shown in Fig. S6.† The peaks at 530 and 531.7 eV can be assigned to the lattice oxygen and oxygen vacancies, respectively.^30^ Sn–Zn–O_*x*_ showed a lower binding energy and an enlarged peak area of oxygen vacancies compared with ZnO and SnO_2_. The increased defect degree could improve eCO_2_RR activity.^31^ The X-ray absorption near-edge structure (XANES) spectra of Sn K-edge and Zn K-edge were obtained and are shown in Fig. 1G and H. The Sn absorption edge of Sn–Zn–O_*x*_ was analogous to the curve of SnO_2_, while a slight negative shift of the absorption edge position compared to SnO_2_ indicates a lower oxidation state of Sn in Sn–Zn–O_*x*_. Meanwhile, the Zn absorption-edge showed an opposite shift compared with ZnO, which revealed the electron transfer from Zn to Sn in Sn–Zn–O_*x*_.^32^ These results are in agreement with the XPS data.

**Fig. 2:**
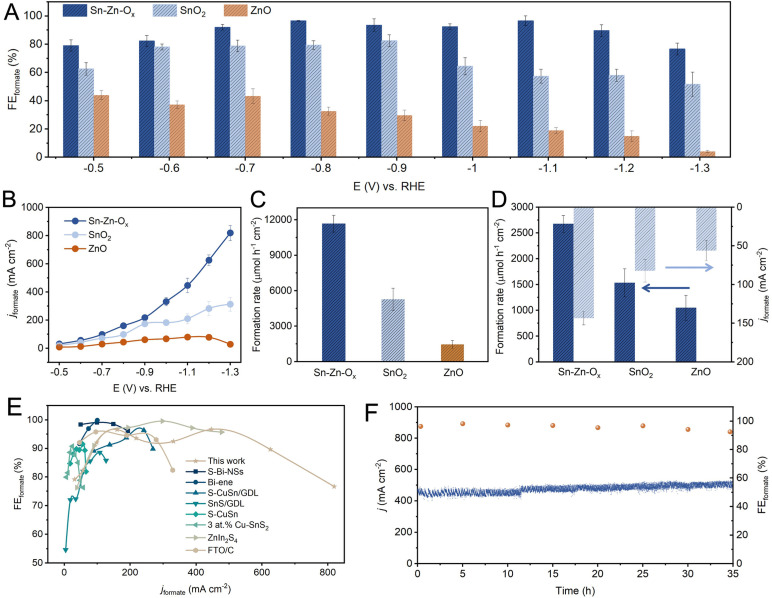
eCO_2_RR performances. (A) The FE_formate_ and (B) *j*_formate_ at different applied potentials on Sn–Zn–O_*x*_, SnO_2_ and ZnO. (C) The formation rates of formate at −1.1 V *vs.* RHE. (D) The ECSA-normalized formate formation rate and partial current density of Sn–Zn–O_*x*_, SnO_2_, and ZnO catalysts at −1.1 V *vs.* RHE. (E) Comparison of FE_formate_ and *j*_formate_ on various reported catalysts. (F) Stability test of Sn–Zn–O_*x*_ at −1.1 V *vs.* RHE.

The eCO_2_RR performances were investigated in a flow cell using 1 M KOH as electrolyte. The gaseous and liquid products were analyzed by gas chromatography (GC) and ^1^H nuclear magnetic resonance (NMR) spectroscopy, respectively. Formate was the only liquid product and its FEs at different potentials are shown in Fig. 2A. The FE_formate_ could be maintained above 90% over Sn–Zn–O_*x*_ in a wide potential window of −0.7 to −1.2 V *vs.* the reversible hydrogen electrode (RHE), and a maximum FE_formate_ of 96.6% can be achieved at −0.8 V *vs.* RHE. However, the maximum FE_formate_ of the as-synthesized SnO_2_ and ZnO was only 79.4% and 43.4%, and H_2_ and CO were detected as the by-products (Fig. S7–S11†).

The partial current density of formate was plotted and is shown in Fig. 2B. A high *j*_formate_ of 625.4 mA cm^−1^ was achieved over Sn–Zn–O_*x*_ at −1.2 V *vs.* RHE with a high FE_formate_ above 90%, which is much higher than that of SnO_2_ and ZnO. When *j*_formate_ increased to 819.6 mA cm^−1^, the FE_formate_ still maintained above 76%. Moreover, the Sn–Zn–O_*x*_ catalyst exhibited a high formation rate of formate of 11 667.4 μmol h^−1^ cm^−2^ at −1.2 V *vs.* RHE, which was 2.2 and 8.0 times higher than that of SnO_2_ and ZnO, respectively (Fig. 2C). The electrochemically active surface area (ECSA) was further assessed according to the double-layer capacitance (*C*_dl_) (Fig. S12 and S13†). As shown in Fig. 2D, the ECSA-normalized *j*_formate_ and formate formation rates were calculated and the value over the Sn–Zn–O_*x*_ catalyst was still the highest, indicating its high intrinsic activity. The attained activity of CO_2_-to-formate can be competitive with those of the best catalysts reported, as the high FE_formate_ and large *j*_formate_ were both available over Sn–Zn–O_*x*_ (Fig. 2E). In addition, an average FE_formate_ of >90% was maintained during continuous electrolysis for 35 h at −1.1 V *vs.* RHE, demonstrating the long-term stability of the Sn–Zn–O_*x*_ catalyst (Fig. 2F).

To directly relate the enhanced selectivity of formate to the influence of the Zn component in the Sn–Zn–O_*x*_ catalyst, Zn(ii) species was selectively removed from Sn–Zn–O_*x*_ by the acid-washing method and used for comparison. After the acid-washing process for 1 h, the Sn/Zn atomic ratio was increased to 4.35. The truncated cubic morphology was still maintained without obvious structural collapse, and Sn, Zn, and O elements were dispersed evenly in the sample (Fig. S14†). However, the sample after removal of Zn species showed much lower CO_2_-to-formate performance than Sn–Zn–O_*x*_ (Fig. S15–S17†), indicating the critical role of Zn species in the Sn–Zn–O_*x*_ catalyst.

**Fig. 3:**
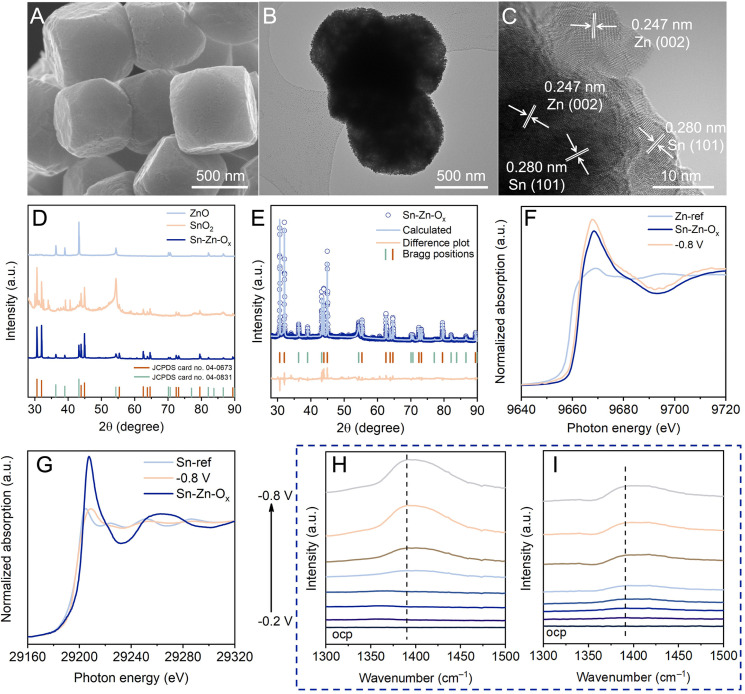
The structural evolution of the catalysts during the eCO_2_RR. (A) SEM image, (B) TEM image, and (C) HR-TEM image of Sn–Zn–O_*x*_ after the eCO_2_RR. (D) Quasi-*in situ* XRD pattern after the eCO_2_RR. (E) Rietveld refinement of the Sn–Zn–O_*x*_ catalyst after the eCO_2_RR using the XRD pattern in (D). Blue circle marks, blue solid line, and light peach solid line represent observed intensities, Rietveld-fit profile, and difference plot, respectively. The sea green and orange tick marks show the positions of the Bragg reflections. (F) Zn K-edge and (G) Sn K-edge XANES spectra of Sn–Zn–O_*x*_ before and after the eCO_2_RR at −0.8 V *vs.* RHE. *In situ* attenuated total reflection surface-enhanced infrared absorption spectroscopy (ATR-SEIRAS) spectra of (H) Sn–Zn–O_*x*_ and (I) SnO_2_.

The structural evolution during the eCO_2_RR was investigated to gain insight into eCO_2_RR enhancement. As revealed by SEM and TEM images (Fig. 3A and B), the catalyst maintained the truncated cubic morphology without obvious structural collapse. Sn, Zn, and O elements still existed and were dispersed evenly in Sn–Zn–O_*x*_ after the eCO_2_RR (Fig. S18†). The HRTEM image displays clear lattice spacings of Sn(101) and Zn(002) planes,^27,33^ indicating the reduction of Sn–Zn–O_*x*_ during the eCO_2_RR (Fig. 3C). The diffraction peaks of Sn (JCPDS 04-0673) and Zn (JCPDS 04-0831) could be detected from quasi-*in situ* XRD measurement (Fig. 3D). According to the Rietveld refinement analysis of the XRD data, the contents of Sn (JCPDS 04-0673) and Zn (JCPDS 04-0831) were estimated to be 85.28% and 14.72% in Sn–Zn–O_*x*_ after the eCO_2_RR (Fig. 3E). From this apparent difference in the content of the two phases, it could be speculated that oxidized Sn exhibited a greater reduction degree than oxidized Zn. Further investigation of the structural evolution was conducted by *in situ* XANES to eliminate interference from air oxidation (Fig. S19†). At the applied potential, the Zn K-edge was shifted to lower energy located between that of the Zn foil (Zn^0^) reference and Sn–Zn–O_*x*_ (Fig. 3F). After the eCO_2_RR, similar features to Sn were detected, where a lower-energy shift of the Sn absorption edge was observed in Sn–Zn–O_*x*_ (Fig. 3G), implying a slightly lower valence state of Sn in Sn–Zn–O_*x*_ compared to Sn foil.^27,34^ According to the above results, the Sn oxides in Sn–Zn–O_*x*_ was reduced to metallic Sn during eCO_2_RR. However, the change in the oxidation state of Zn was relatively small, resulting in more electron accumulation on Sn. It contributes to CO_2_ activation and HCOO* intermediate adsorption, leading to enhanced eCO_2_RR performance.

*In situ* ATR-SEIRAS measurements were performed to monitor possible reaction intermediates. According to Fig. 3H and I, the IR band at 1390 cm^−1^ associated with O–C–O vibration in the bidentate HCOO* intermediate was monitored,^35,36^ and its intensities increased with the increasing potential. This is in agreement with the trend in formate formation rates. Moreover, the band intensity of HCOO* over Sn–Zn–O_*x*_ was stronger than that over SnO_2_. This phenomenon is consistent with the results of CO_2_-to-formate performance, implying that the HCOO* intermediate was the main factor in the generation of formate.^37^ The sharp contrast suggested that the introduction of Zn played an important role in promoting the HCOO* intermediate production.^35^

The dissociation of H_2_O in an alkaline environment is a sluggish step, which can be detrimental to the PECT processes during the eCO_2_RR to formate. Therefore, a catalyst with optimal water dissociation is required to ensure the proton-feeding rate in the eCO_2_RR to formate. As shown in Fig. S20,† a negative IR band at 1630 cm^−1^ ascribed to adsorbed H_2_O was detected.^38^ The band intensity of Sn–Zn–O_*x*_ was stronger than that of SnO_2_, indicating that the introduction of Zn could accelerate the activation of H_2_O. As the cathodic potential was applied, H_2_O molecules underwent activation to yield protons for the further protonation of *CO_2_ to form the HCOO* intermediate, which was confirmed using a stronger IR band for the HCOO* intermediate. These results illustrated that Sn–Zn–O_*x*_ favored the formation and stabilization of the HCOO* intermediate, which contributed to the enhanced eCO_2_RR performance.

**Fig. 4:**
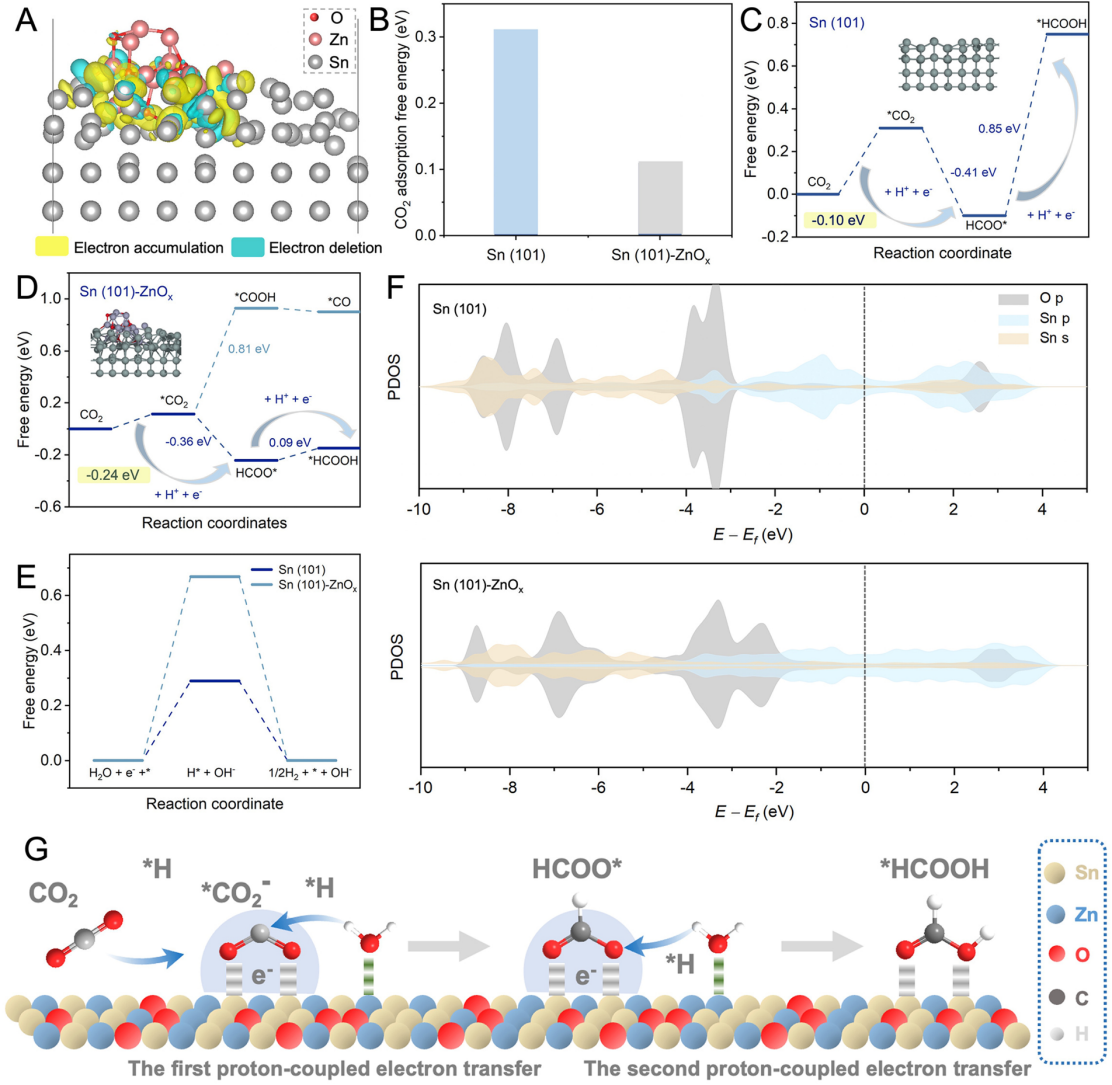
DFT calculations and eCO_2_RR mechanism discussion. (A) Schematic of calculated charge densities among Sn and Zn atoms. (B) CO_2_ adsorption free energy. (C) Gibbs free-energy diagrams of the eCO_2_RR to HCOOH on Sn(101). (D) Gibbs free-energy diagrams of the eCO_2_RR to HCOOH and CO on Sn(101)–ZnO_*x*_. (E) Gibbs free-energy diagrams for the HER process on Sn(101) and Sn(101)–ZnO_*x*_. (F) PDOS of the p orbital of the O atom and s and p orbitals of the Sn atom on the Sn(101) and Sn(101)–ZnO_*x*_ models with adsorbed HCOO*. (G) Schematic of the eCO_2_RR-to-HCOOH catalytic mechanism over the Sn–Zn–O_*x*_ catalyst.

In addition, DFT calculations were performed to elucidate the mechanism for enhanced activity and selectivity of the eCO_2_RR. According to the catalyst characterization data and structural optimization, Sn(101) and Sn(101)–ZnO_*x*_ models were constructed to represent SnO_2_ and Sn–Zn–O_*x*_, respectively. The detailed data about the computational structure models and relevant parameters are shown in Fig. S21–S24.† The electronic structure and interactions of Sn(101)–ZnO_*x*_ were investigated using the calculated charge density distribution. As shown in Fig. 4A, the charge density was depleted around Zn atoms and accumulated around Sn atoms, revealing the electron transfer from Zn atoms to Sn atoms and resulting in electron-rich Sn atoms. CO_2_ binding capability is a prerequisite for the eCO_2_RR. As shown in Fig. 4B, the CO_2_ adsorption free energy on Sn(101)–ZnO_*x*_ was much lower than that on Sn(101), which was in agreement with the results of the CO_2_ adsorption isotherms in Fig. S25.† The above results indicate preferable CO_2_ adsorption on electron-rich Sn in the Sn–Zn–O_*x*_ catalyst.

Fig. 4C displays the Gibbs free energy profiles for the pathway of the eCO_2_RR to formate on Sn(101). As the first step, the CO_2_ activation process (CO_2_ → *CO_2_) is essential for the formation of the key intermediate HCOO* in the eCO_2_RR to formate.^39,40^ The formation of *CO_2_ on Sn (101) was endergonic, and the high free energy of *CO_2_ formation (0.31 eV) was not conducive to HCOO* generation. By contrast, Sn(101)–ZnO_*x*_ showed a lower energy barrier (0.11 eV) for *CO_2_ formation (Fig. 4D), which was favorable for the subsequent hydrogenation reaction to form the HCOO* intermediate. This makes the free-energy step involved in the first PECT toward formate formation more thermodynamically accessible for Sn(101)–ZnO_*x*_. The process of HCOO* undergoing the second PECT to form *HCOOH was the rate-determining step (RDS) for the HCOOH pathway on Sn(101). The Gibbs free energy for this RDS was found to be up to 0.85 eV. Sn(101)–ZnO_*x*_ could effectively reduce the free energy of *HCOOH formation to 0.09 eV and convert the RDS into *CO_2_ → HCOO*. The changed RDS pathway led to a decrease in the energy barrier for HCOOH formation on Sn(101)–ZnO_*x*_. These results indicate that the Sn–Zn–O_*x*_ catalyst with electron-rich Sn enabled a promotion in the formation of formate compared to SnO_2_. Furthermore, Sn(101)–ZnO_*x*_ presented a significantly higher free energy barrier for *COOH formation than for HCOO* formation, suggesting that the HCOOH pathway was more thermodynamically favorable than the CO pathway.^41^ This clarified the high selectivity of Sn(101)–ZnO_*x*_ toward formate formation. In addition, Sn(101)–ZnO_*x*_ showed a higher energy barrier for the generation of *H intermediates compared to Sn(101) (Fig. 4E), indicating that the HER was inhibited on Sn(101)–ZnO_*x*_.

To further elucidate the promoting effect of Sn(101)–ZnO_*x*_, the projected density of states (PDOS) was analyzed to explore the interaction between the O atoms in key intermediate HCOO* and the Sn atoms on catalyst models. As illustrated in Fig. 4F, Sn(101)–ZnO_*x*_ shows more harmonic p–p and p–s overlaps between the O 2p and Sn 5s and 5p orbitals than Sn(101), indicating the enhancement of interactions between the active site and HCOO* intermediate after the introduction of Zn.^22^ In addition, the upshift of the O 2p orbital away from the Fermi level (*E*_f_) suggests an increased antibonding state of the O atom in absorbed HCOO* on Sn(101)–ZnO_*x*_ compared to that on Sn(101).^42,43^ This means that HCOO* absorbed on Sn(101)–ZnO_*x*_ enables H* to adsorb and react with it more accessibly, leading to a decline in the Gibbs free energies of the PECT process for the formation of HCOOH. Based on the discussion above, the catalytic mechanism of Sn–Zn–O_*x*_ for the eCO_2_RR was outlined and is shown in Fig. 4G. First, electron-rich Sn could promote the adsorption and activation of CO_2_ molecules to generate *CO_2_. Meanwhile, the positive-valence Zn sites were more likely to drag the O atom in the absorbed H_2_O, which might promote the combination of H* and carbonaceous intermediates in the PCET process. Then, the lower energy barriers for the formation of HCOO* and *HCOOH are conducive to *CO_2_ → HCOO* → *HCOOH proceeding rapidly. Moreover, electron-rich Sn electrocatalysts induced by Zn species in Sn–Zn–O_*x*_ might suppress H_2_ evolution. As a consequence, the rationally constructed electron-rich Sn catalyst achieved high catalytic activity and excellent selectivity for the eCO_2_RR to formate.

## Conclusions

In summary, Sn–Zn–O_*x*_ has been successfully prepared and used as an efficient electrocatalyst for CO_2_-to-formate. The highest FE_formate_ of 96.6% could be achieved and it can maintain a high FE_formate_ above 90% at *j*_formate_ up to 625.4 mA cm^−1^. The *in situ* experimental results demonstrated the structural evolution of the catalysts and their significant role in improving the eCO_2_RR-to formate performance. The accumulation of electron density around Sn facilitates the activation of CO_2_ molecules to form a 
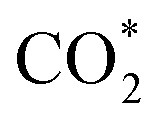
 intermediate, which is conducive to the formation HCOO* species. Moreover, Sn–Zn–O_*x*_ can modulate the adsorption configuration of HCOO* by increasing the antibonding state of the O atom in absorbed HCOO*, thereby lowering the energy barrier for the PECT for HCOO* → HCOOH* and facilitating CO_2_-to-formate conversion. This work offers an effective strategy that coupled electronic structure manipulation and intermediate optimization for CO_2_ electroreduction to formate.

## Data availability

All experimental data is available in the ESI.†

## Author contributions

X. X. T. performed all the experiments. S. H. J., X. N. S., X. D. M., J. Q. F, L. B. Z., and L. M. W. performed the analysis of the experimental data. J. D., A. B. C. and Q. G. Z. participated in discussions. X. F. S. and B. X. H. co-supervised the whole project. All authors discussed the results and commented on the manuscript.

## Conflicts of interest

The authors declare no competing financial interests.

## Supplementary Material

SC-014-D3SC02790B-s001
