# Supplementary material for: Zn-induced electron-rich Sn catalysts enable highly efficient CO2 electroreduction to formate
Source: Chem Sci. 2023 Jul 10;14(30):8214–21. doi: 10.1039/d3sc02790b (PMC10395268; doi:10.1039/d3sc02790b)
Supplement: SC-014-D3SC02790B-s001 [file SC-014-D3SC02790B-s001.pdf]

Electronic Supplementary Material (ESI) for Chemical Science.
This journal is © The Royal Society of Chemistry 2023

Electronic Supplementary Information

Zn-Induced Electron-Rich Sn Catalysts Enable Highly Efficient CO_2 Electroreduction to Formate

Xingxing Tan[1,2], Shunhan Jia[1,2], Xinning Song[1,2], Xiaodong Ma[1,2], Jiaqi Feng[1], Libing Zhang[1,2], Limin Wu[1,2], Juan Du[3], Aibing Chen[3], Qinggong Zhu[1,2], Xiaofu Sun*[1,2], and Buxing Han*[1,2,4]

[1] Beijing National Laboratory for Molecular Sciences, Key Laboratory of Colloid and Interface and Thermodynamics, Center for Carbon Neutral Chemistry, Institute of Chemistry, Chinese Academy of Sciences, Beijing 100190, China; E-mail: sunxiaofu@iccas.ac.cn; hanbx@iccas.ac.cn

[2] School of Chemical Sciences, University of Chinese Academy of Sciences, Beijing 100049, China

[3] College of Chemical and Pharmaceutical Engineering, Hebei University of Science and Technology, Shijiazhuang 050018, China

[4] Shanghai Key Laboratory of Green Chemistry and Chemical Processes, School of Chemistry and Molecular Engineering, East China Normal University, Shanghai 200062, China.

Experimental Section

Materials

Stannic chloride pentahydrate ($SnCl_4 \cdot 5H_2O$, purity>99%), Zinc(II) chloride ($ZnCl_2$, purity>99%), Sodium hydroxide (NaOH, purity>99%), potassium hydroxide (KOH, ≥ 85%), D_2O (98%), and sodium 2, 2-dimethyl-2-silapentane-5-sulfonate (DSS, 99%) were purchased from Alfa Aesar China Co., Ltd. Ethanol was obtained from Concord Technology (Tianjin) Co., Ltd. Citric acid (purity>99%) was purchased from Beijing Innochem Science & Technology Co. Ltd. Ar (99.999%) and CO_2 (99.999%) were provided by Beijing Analytical Instrument Company. Deionized water was used in the experiments. All the chemicals were used as received.

Preparation of catalysts

The Sn-Zn-O_x catalyst was prepared using a facile coprecipitation method followed by pyrolyzing. Firstly, $SnCl_4 \cdot 5H_2O$ (8.0 mmol) was dissolved in 20 mL ethanol. $ZnCl_2$ (4.0 mmol) and citric acid (4.0 mmol) were dissolved in 140 mL water. The above ethanol solution was added into the $ZnCl_2$ aqueous solution under vigorous stirring to form a mixed solution. Then, 20 mL NaOH (2 M) aqueous solution was added into the above mixed solution dropwise with a constant stirring for 1 hour. Another 30 mL NaOH (2 M) aqueous solution was added into the above suspension dropwise and stirred for additional 30 min. Finally, the sediments were collected and rinsed thrice with deionized water and ethanol, followed by centrifuging and drying under vacuum at 60 °C for 12 h. Under an argon atmosphere, the obtained samples

were annealed at 500 °C for 2 h with a heating rate of 5 °C min^{-1}. The SnO_2 and ZnO were produced by the same method, but without the addition of $ZnCl_2$ or $SnCl_4 \cdot 5H_2O$, respectively. To selectively remove Zn species, the prepared Sn-Zn-O_x sample was dispersed in 1 M HCl for 1 hour at room temperature under stirring. The product was rinsed with deionized water until the solution became neutral, and finally dried at 60 °C.

Characterization

The morphologies of the catalysts were characterized by a HITACHI SU8020 scanning electron microscope (SEM) and a JEOL JEM-2100F high-resolution transmission electron microscopy (HR-TEM). X-Ray diffraction (XRD) analysis of the samples was conducted on a Rigaku D/max-2500 X-ray diffractometer with Cu-Kα radiation and the scan speed was 5° min^{-1}. X-ray photoelectron spectroscopy (XPS) analysis was conducted on the Thermo Scientific ESCA Lab 250Xi using 200 W monochromatic Al Kα radiation and 500 μm X-ray spot. The base pressure in the analysis chamber was about 3×10^{-10} mbar. Typically, the hydrocarbon C1s line at 284.8 eV from adventitious carbon was used for energy referencing. The X-ray adsorption spectroscopy (XAS) measurements were performed using a modified flow cell at the 1W1B beamline at Beijing Synchrotron Radiation Facility (BSRF) and BL14W1 of the Shanghai Synchrotron Radiation Facility (SSRF), China. The data were collected in fluorescence excitation mode using a Lytle detector. Sn foil, SnO, Zn foil, and ZnO were used as references. The acquired EXAFS data were processed according to the standard procedures using the Athena and Artemis implemented in

the IFEFFIT software packages.

Electrode preparation

To construct the cathode electrode, a catalyst slurry containing 5 mg of obtained catalysts, 1 mL of isopropanol and 20 μL of Nafion ionomer solution (5 wt% in H_2O) was first prepared and sonicated for 1 h. Then, the catalyst slurry (0.3 mL) was slowly air-brushed onto the carbon paper with a hydrophobic microporous gas diffusion layer (YLS-30T GDL) under vacuum to achieve a catalyst loading of ~ 1.5 mg cm^{-2}. Ni foam were used as anode electrode.

Electrochemical study

Electrochemical reduction of CO_2 in a flow cell. Electrochemical studies were conducted in an electrochemical flow cell which was composed of a gas chamber, a cathodic chamber, and an anodic chamber. An anion exchange membrane (FumasepFAA-3-PK-130) was used to separate the anodic and cathodic chambers, and a Hg/HgO electrode and Ni foam were used as the reference and counter electrodes, respectively. The electrolysis was conducted using a CHI 660e electrochemical workstation equipped with a high current amplifier CHI 680c. The measured potentials after *i*R compensation were rescaled to the reversible hydrogen electrode (RHE) reference by E (versus RHE) = E (versus Hg/HgO) + 0.098 V+0.0591V/pH × pH. For performance studies, 1 M KOH was used as the electrolyte, and it was circulated through the cathodic and anodic chambers using peristaltic pumps at a rate of 50 mL min^{-1}. The flow rate of CO_2 gas through the gas chamber

was controlled to be 30 sccm using a digital gas flow controller. To maintain the eCO_2RR performance of the catalysts during the stability test, the electrolyte was refreshed timely and continuously.

Double-layer capacitance (*C_{dl}*) measurements. The electrochemical active surface area is proportional to C_{dl} value. C_{dl} was determined by measuring the capacitive current associated with double-layer charging from the scan-rate dependence of cyclic voltammogram (CV). The CV ranged from -0.01 V to -0.11 V vs. Hg/HgO. The C_{dl} was estimated by plotting the Δj (j_a-j_c) at -0.06 V vs. Hg/HgO against the scan rates, in which the j_a and j_c are the anodic and cathodic current density, respectively. The scan rates were 20, 40, 60, 80, 100 and 120 mV s^{-1}.

In-situ attenuated total reflection-surface-enhanced IR absorption spectroscopy (ATR-SEIRAS) measurement.

The ATR-SEIRAS experiments were conducted in a modified electrochemical cell that integrated into a Nicolet 6700 FTIR spectrometer equipped with mercury-cadmium-telluride (MCT) detector cooled by liquid nitrogen. The catalysts ink was dropped on a germanium (Ge) ATR crystal deposited with Au film. A carbon rod and an Ag/AgCl electrode were used as counter and reference electrodes. Each spectrum was collected with a time resolution of 42 s, Before the test, the initial state with open circuit was scanned for a background. All applied potentials were converted to the reversible hydrogen electrode (RHE).

Product analysis. The gaseous product of electrochemical experiments was collected using a gas bag and analyzed by gas chromatography (GC Agilent 8890), which was equipped with TCD detectors using argon as the carrier gas. The liquid product was analyzed by ^{1}H NMR (Bruker Avance III 400 HD spectrometer) in deuteroxide with phenol and sodium 2, 2-dimethyl-2-silapentane-5-sulfonate (DSS) as internal standards.

Calculations of Faradaic efficiencies of gaseous and liquid products

Gaseous products:

From the GC peak areas and calibration curves for the TCD detector,the V % of gaseous products were obtained. Since the flow rate of the CO_2 was constant, the moles of gaseous products can be calculated.

The Faradaic efficiency of gaseous product is:

$$FE = \frac{moles\ of\ product}{Q/nF} \times 100\%$$

(Q: charge (C); F: Faradaic constant (96485 C mol^{-1}); n: the number of electrons required to generate the product)

Liquid products:

After electrolysis, a certain amount of internal standard solution was added to the electrolyte as the internal standard. Because the concentration of internal standard was known, the moles of liquid products can be calculated from integral areas and

calibration curves. To accurately integrate the products in NMR analysis, two standards located in different regions were used in NMR analysis. The DSS was the reference for ethanol, and the phenol was the reference for formate. 400 μL catholyte after the electrocatalytic CO_2 reduction reaction was mixed with 100 μL 6 mM DSS solution, 100 μL 200 mM phenol and 200 μL D_2O, and then analyzed by ^{1}H NMR (Bruker Advance III 400 HD spectrometer).

The Faradaic efficiency of liquid product is:

$$FE = \frac{moles\ of\ product}{Q/nF} \times 100\%$$

(Q: charge (C); F: Faradaic constant (96485 C mol^{-1}); n: the number of electrons required to generate the product)

Computational Methods

Spin-polarized density functional theory (DFT) calculations were performed with the Vienna ab initio simulation package (VASP).[1] The projector-augmented-wave (PAW) pseudopotential was used for core electrons, a cutoff energy of 450 eV was sued for valence electrons, and the generalized gradient approximation (GGA) in the form of Perdew−Burke−Ernzerhof (PBE) was used for the exchange-correlation potentials.[2,3] To avoid interactions between adjacent images and explore many materials space, the lateral sizes of constructed models are around 18.58 and 19.71 Å, and the *c*-axis was set to be 31.97 Å. The atoms were fully relaxed until the force acting on each atom was less than 0.02 eV/Å, and the energy convergence reached 1×10^{-5} eV. Van der

Waals (vdW) interaction was considered at the DFT-D3 level as proposed by Grimme with the Becke-Jonson damping method.[4]

The computational hydrogen electrode model (CHE) was used to simulate the electrochemical reduction of CO_2, which shows that the Gibbs free energy of proton-electron pair ($H^+ + e^-$) is a half of the Gibbs free energy of H_2 at potential of 0 V.[5] The Gibbs free energy of each reaction species was computed as $G = E + E_{ZPE} - TS$, where E is the total energy from the DFT calculation, while E_{ZPE} and TS are the zero-point energy and entropy contributions of adsorbates obtained from vibration analysis aided by VASPKIT package.[6]

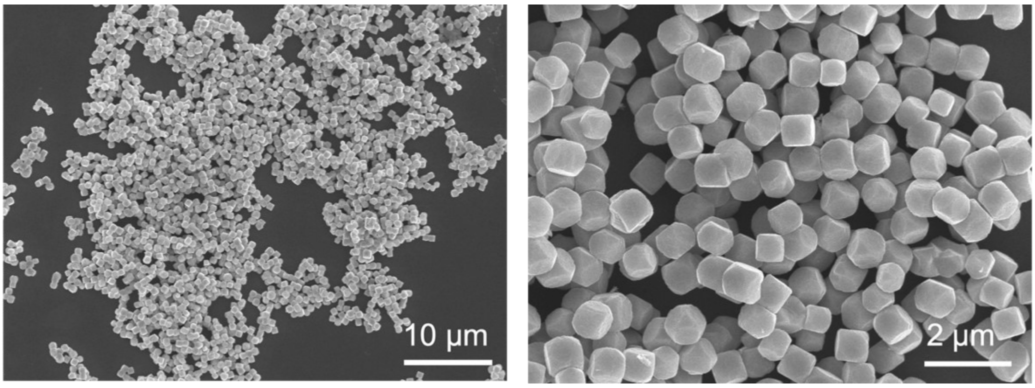

Figure S1. The SEM images of Sn-Zn-O_x.

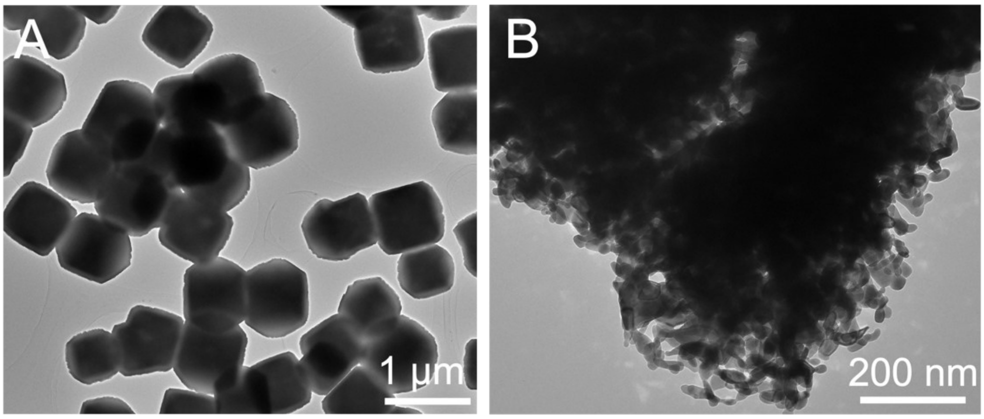

Figure S2. The TEM images of Sn-Zn-O_x.

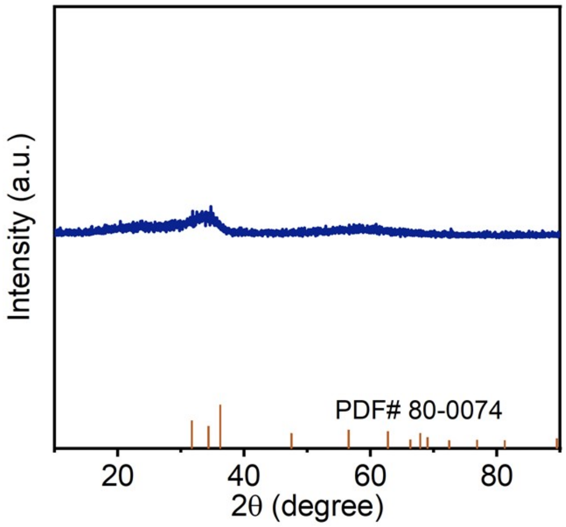

Figure S3. XRD pattern of Sn-Zn-O_x.

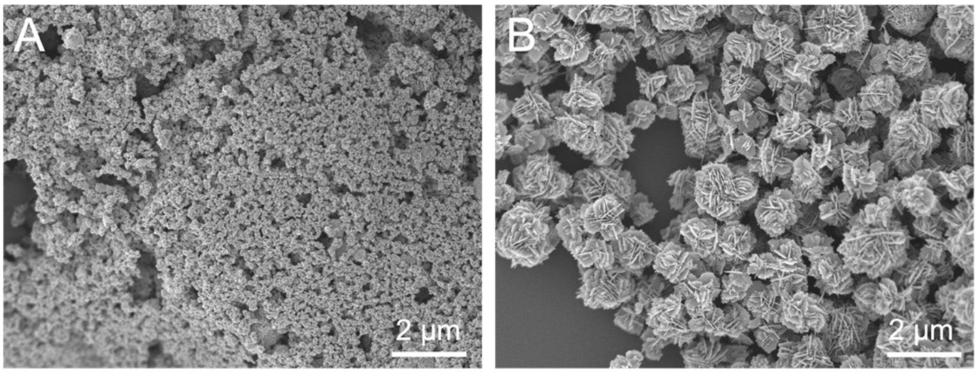

Figure S4. The SEM images of (A) SnO_2 and (B) ZnO.

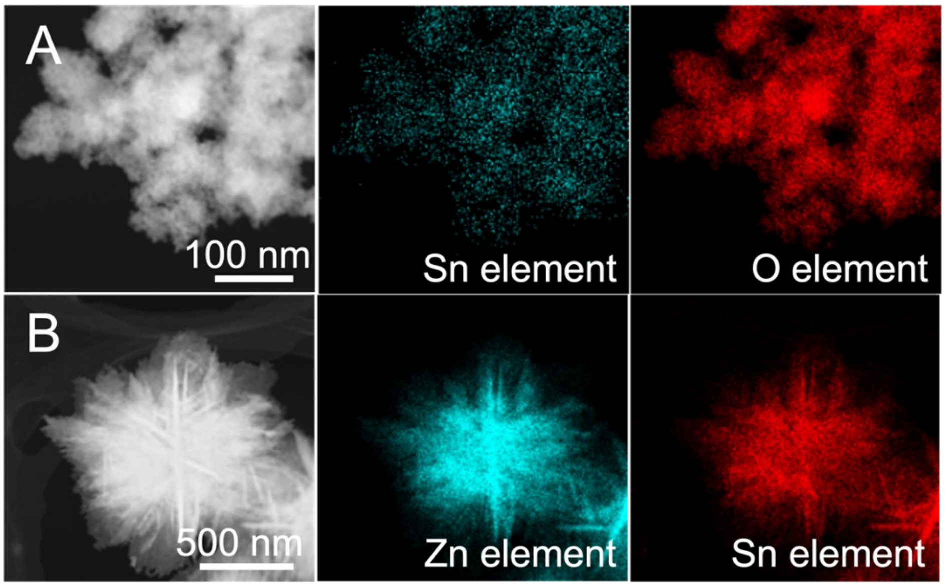

Figure S5. Energy-dispersive X-ray spectroscopy of (A) SnO_2 and (B) ZnO.

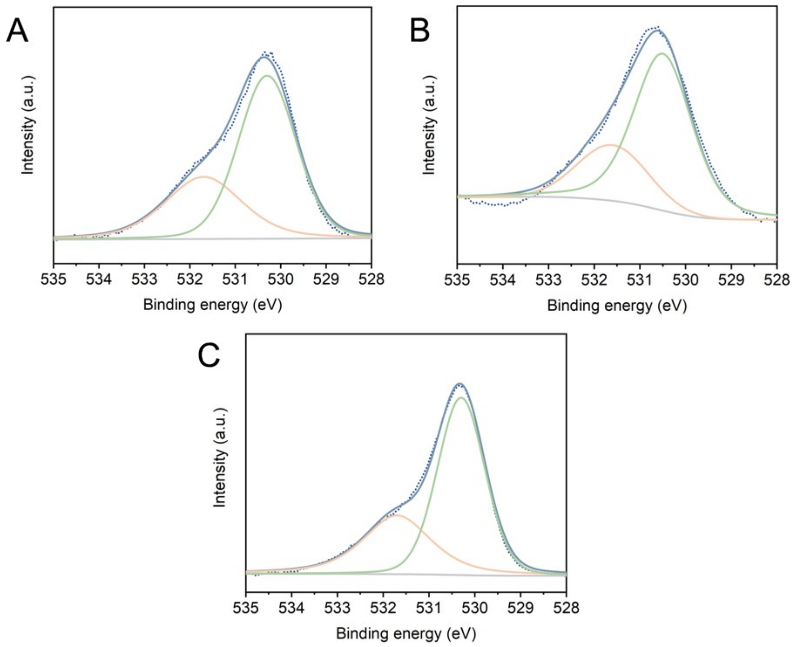

Figure S6. O 1s XPS spectra for (A) Sn-Zn-O_x, (B) SnO_2, and (C) ZnO.

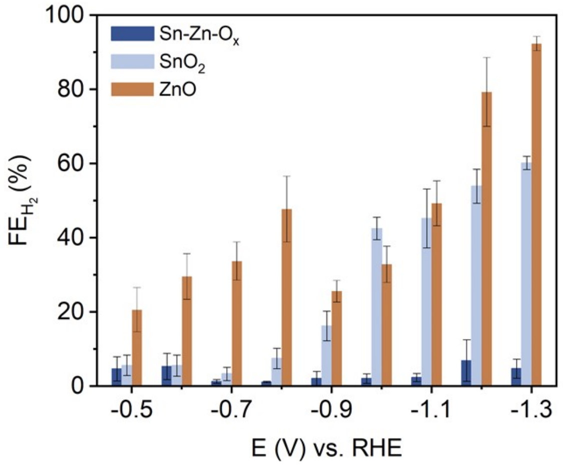

Figure S7. The FE of H_2 under different applied potentials over Sn-Zn-O_x, SnO_2, and ZnO, catalysts.

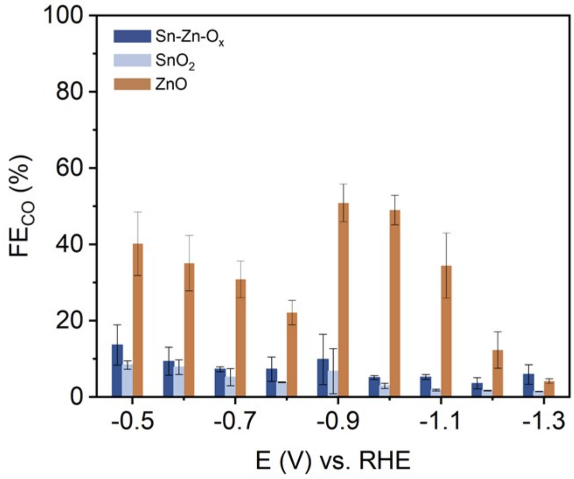

Figure S9. The FE_{CO} under different applied potentials over Sn-Zn-O_x, SnO_2, and ZnO catalysts.

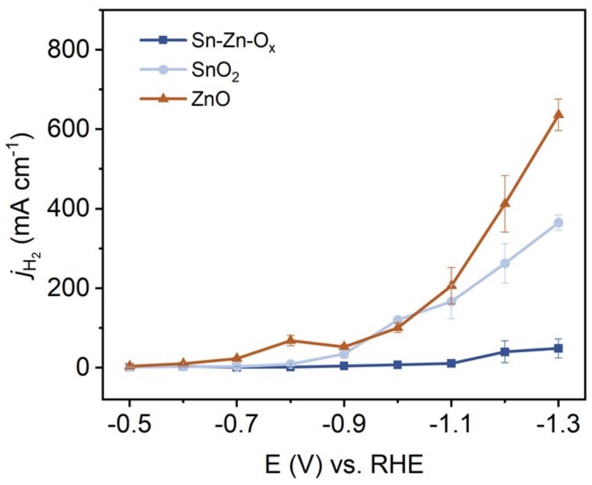

Figure S10. The partial current densities of H_2 under different applied potentials over Sn-Zn-O$_x$, SnO_2, and ZnO catalysts.

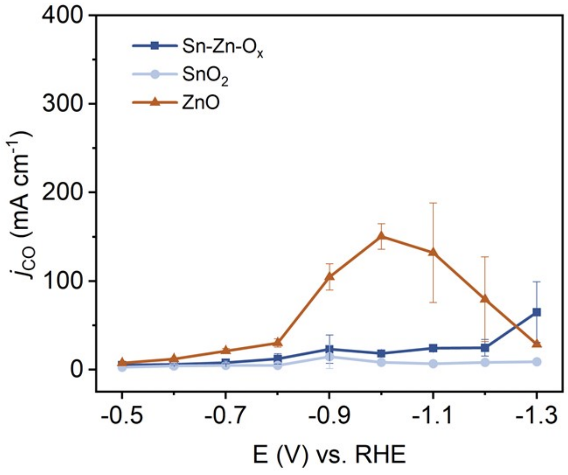

Figure S11. The partial current densities of CO under different applied potentials over Sn-Zn-O$_x$, SnO_2, and ZnO catalysts.

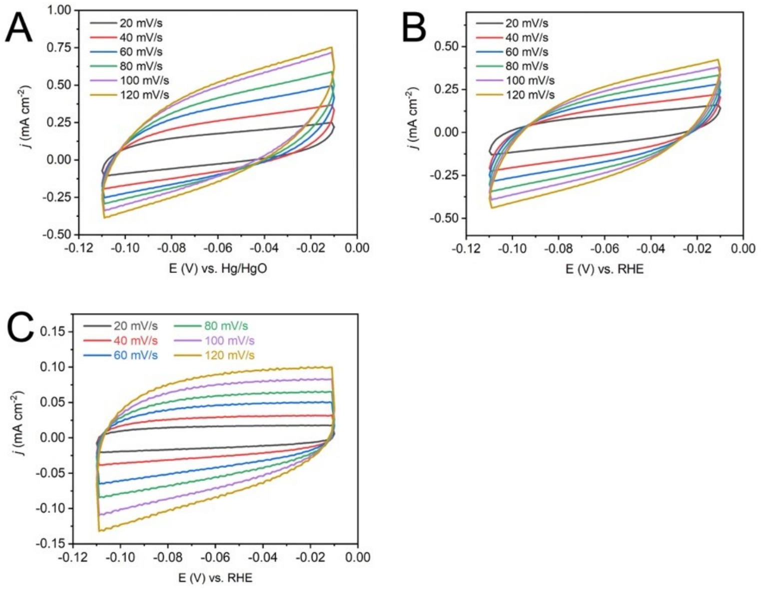

Figure S12. The cyclic voltammetry at different scan rates (20, 40, 60, 80, 100, 120 mV s^{-1}) over Sn-Zn-O_x, SnO_2, and ZnO catalysts.

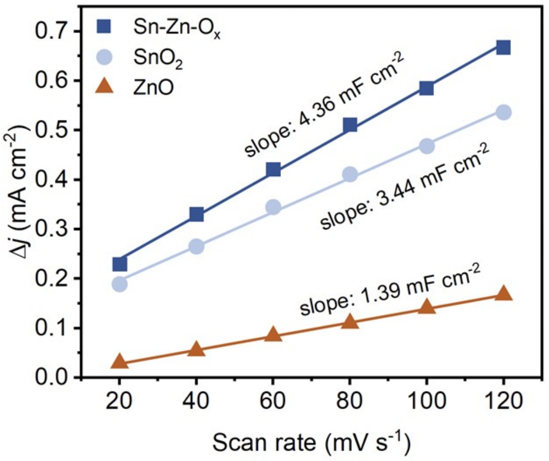

Figure S13. Charging current density differences plotted against scan rates.

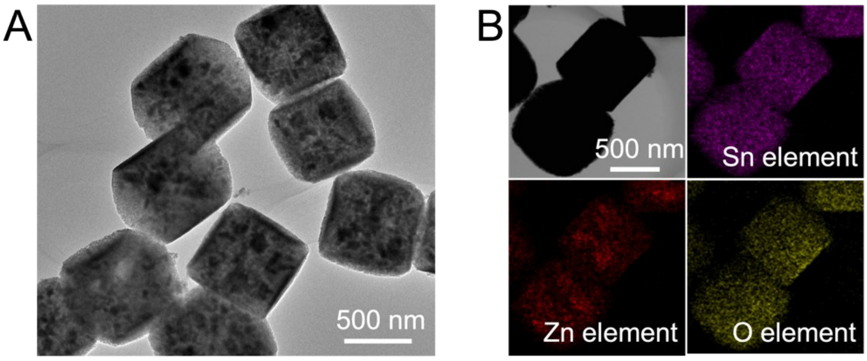

Figure S14. (A) The TEM image and (B) energy-dispersive X-ray spectroscopy of Sn-Zn-O_x after removing Zn.

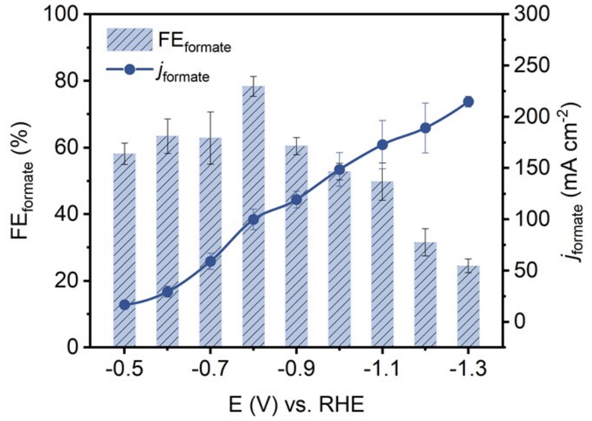

Figure S15. The $FE_{formate}$ and $j_{formate}$ at different applied potentials on Sn-Zn-O_x after acid-washing to selectively remove Zn (II) species.

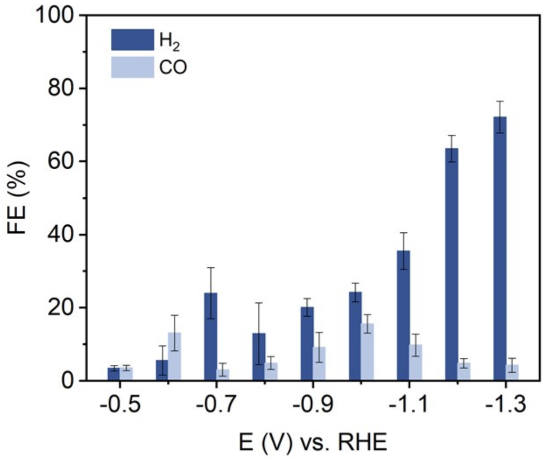

Figure S16. The FE of H_2 and CO under different applied potentials on Sn-Zn-O_x after acid-washing to selectively remove Zn (II) species.

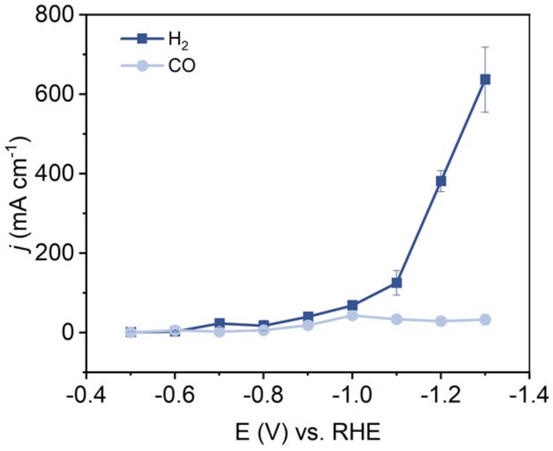

Figure S17. The partial current densities of H_2 and CO under different applied potentials on Sn-Zn-O_x after acid-washing to selectively remove Zn (II) species.

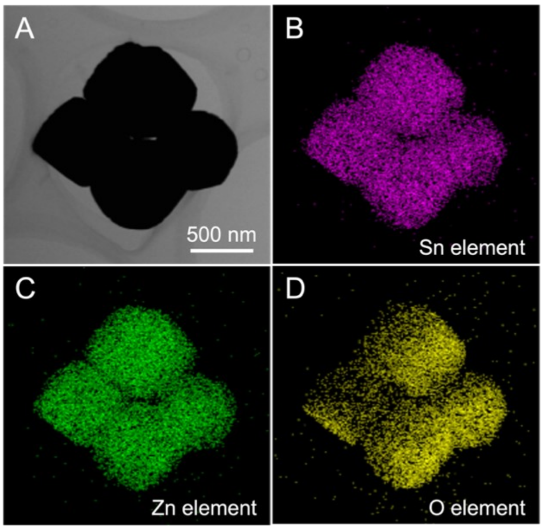

Figure S18. Energy-dispersive X-ray spectroscopy of Sn-Zn-O_x after eCO_2RR.

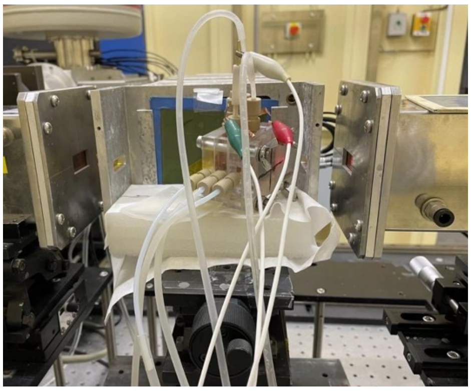

Figure S19. *In situ* electrochemical cell for XAS test.

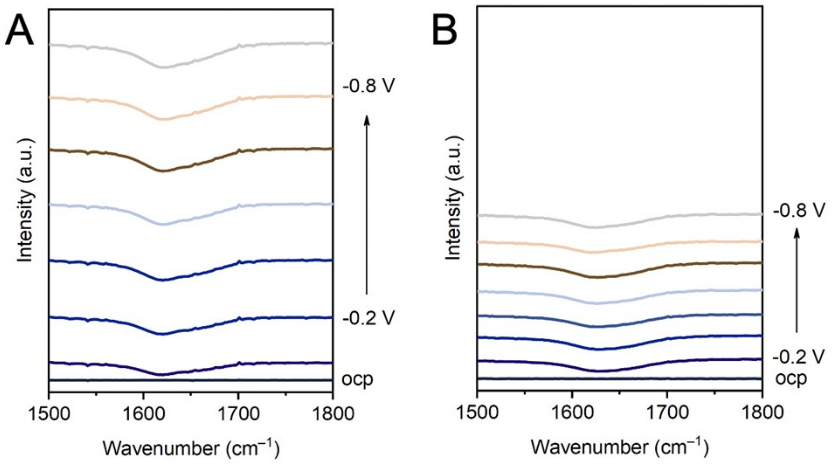

Figure S20. *In situ* ATR- SEIRAS spectra of (A) Sn-Zn-O_x and (B) SnO_2.

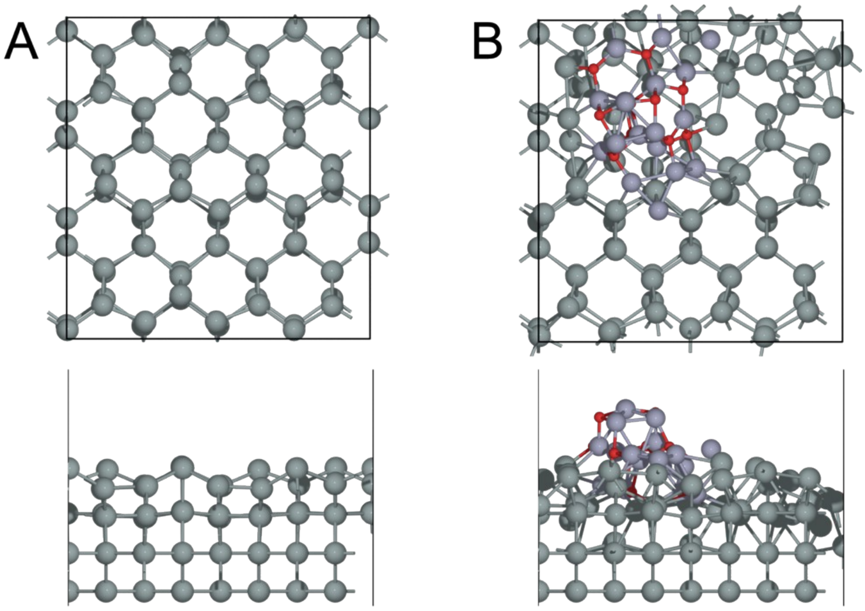

Figure S21. Well optimized substrates of (A) Sn(101) and (B) Sn(101)-ZnO_x.

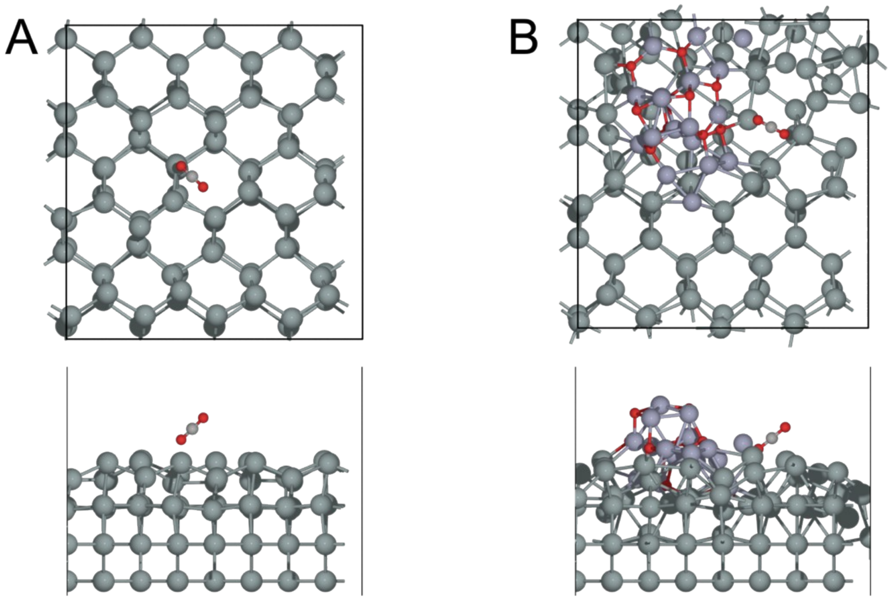

Figure S22. The optimal configurations of adsorbed CO_2 on (A) Sn(101) and (B) Sn(101)-ZnO_x.

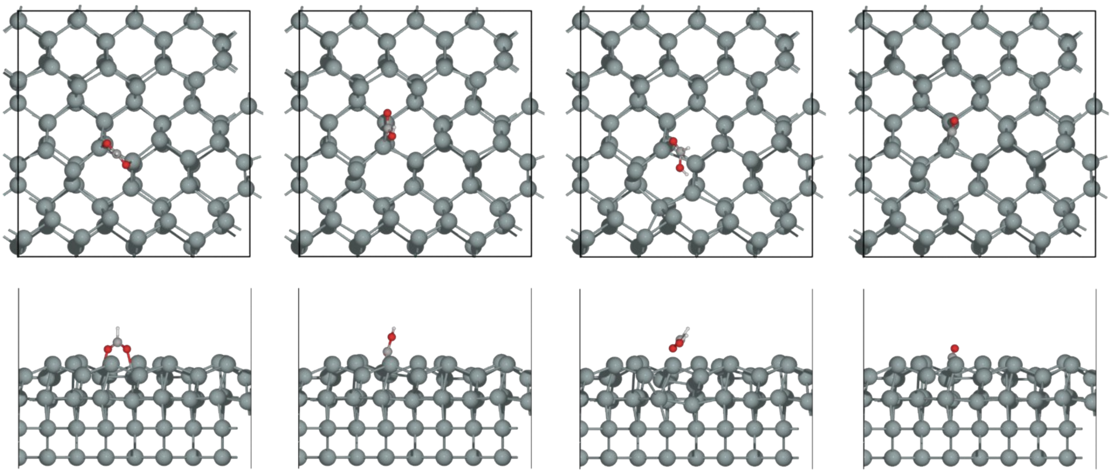

Figure S23. The catalytic pathway of CO_2-to-HCOOH on Sn(101) according to the optimized configurations with adsorbed intermediates.

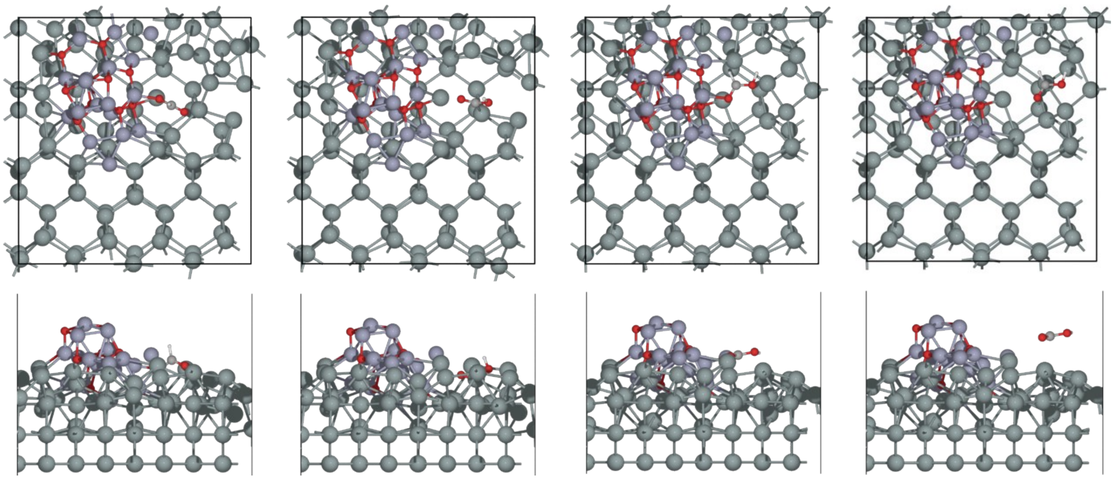

Figure S24. The catalytic pathway of CO_2-to-HCOOH on Sn(101)-ZnO_x according to the optimized configurations with adsorbed intermediates.

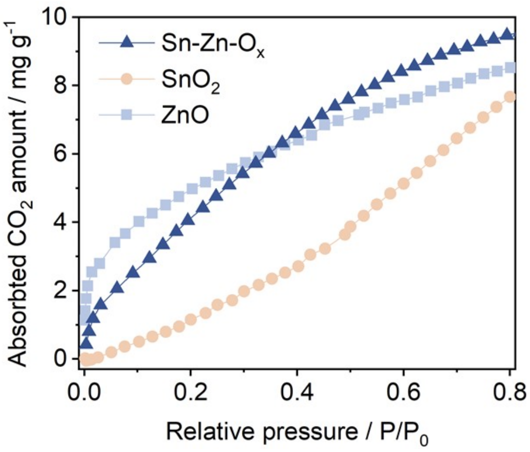

Figure S25. The CO_2 adsorption isotherms at 25 °C on Sn-Zn-O_x, SnO_2, and ZnO.

References

( ) Kresse, G.; Furthmüller, J. Efficiency of ab-Initio Total Energy Calculations for Metals and Semiconductors Using a Plane-Wave Basis Set. *Comput. Mater. Sci.* **1996**, 6, 15-50.

(2) Blöchl, P. E. Projector Augmented-Wave Method. Phys. Rev. B: *Condens. Matter Mater. Phys.* **1994**, 50, 17953.

(3) Perdew, J. P.; Burke, K.; Ernzerhof, M. Generalized Gradient Approximation Made Simple. *Phys. Rev. Lett.* **1996**, 77, 3865-3868.

(4) Grimme, S. Semiempirical GGA-type density functional constructed with a long-range dispersion correction. *J. Comput. Chem.* **2006**, *2*7, 1787-1799.

(5) Nørskov, J. K.; Rossmeisl, J.; Logadottir, A.; Lindqvist, L.; Kitchin, J. R.; Bligaard, T.; Jónsson, H. Origin of the Overpotential for Oxygen Reduction at a Fuel-Cell Cathode. *J. Phys. Chem. B* **2004**, 108, 17886-17892.

(6) Wang, V.; Xu, N.; Liu, J.-C.; Tang, G.; Geng, W.-T. VASPKIT: A user-friendly interface facilitating high-throughput computing and analysis using VASP code. *Comput. Phys. Commun.* **2021**, 267, 108033.
